# pymgpipe: microbiome metabolic modeling in Python

**DOI:** 10.21105/joss.05545

**Published:** 2023-08-02

**Authors:** Yoli Meydan, Federico Baldini, Tal Korem

**Affiliations:** 1 Program for Mathematical Genomics, Department of Systems Biology, Columbia University Irving Medical Center, New York, NY, United States of America.; 2 Department of Obstetrics and Gynecology, Columbia University Irving Medical Center, New York, NY, United States of America.

## Introduction

Microbially-produced metabolites and microbiome metabolism in general are strongly linked to ecosystem-level phenotypes, including the health of the human host (Bar et al., 2020; Villanueva-Millán et al., 2015). To aid in the study of microbial metabolism from observational, human-derived data, a variety of computational methods that predict microbial community metabolic output from microbial abundances have been developed (Baldini et al., 2019; Diener et al., 2020; Mallick et al., 2019; Noecker et al., 2022). Several of these methods rely on community-scale metabolic models, which are mechanistic, knowledge-based models that enable the formulation and *in silico* testing of biological hypotheses regarding the metabolism of microbial communities (Baldini et al., 2019; Diener et al., 2020). Community-scale models primarily use Flux Balance Analysis, a technique that infers the metabolic fluxes in a system by optimizing an objective function, typically growth rate, subject to an assumption of a steady state and constraints imposed by the metabolic reactions present in the system (Orth et al., 2010). These metabolic reactions are obtained from genome-scale metabolic networks (GEMs), knowledge-based computational models encompassing the known biochemical reactions present within an organism (Thiele & Palsson, 2010). In recent years, curated GEMs for thousands of human-associated microbial organisms have become increasingly available, enabling a more in-depth exploration of the human microbiome (Heinken et al., 2023; Machado et al., 2018; Norsigian et al., 2020). In addition, several community-scale metabolic modeling methods specifically tailored to the human microbiome have emerged, such as MICOM and mgPipe (Baldini et al., 2019; Diener et al., 2020).

## Statement of need

mgPipe is a method that combines individual GEMs into a shared compartment according to the microbial abundances observed in every sample to construct a community-level metabolic model. Input and output compartments are added to allow for a distinction between the uptake and secretion of metabolites by the community. After constructing a representative model for each sample, mgPipe computes the metabolic capacity for all present metabolites in the form of Net Maximal Production Capacities (NMPCs). NMPCs are calculated as the absolute difference between the maximum secretion through the output compartment and the maximal uptake through the input compartment (Baldini et al., 2019). To accomplish this, Flux Variability Analysis (FVA) (Mahadevan & Schilling, 2003) is used to compute reaction bounds (minimum and maximum fluxes) through metabolite exchange reactions.

mgPipe models can further be used to explore metabolic interactions among individual taxa, the contribution of these taxa to the overall community metabolism, and to raise hypotheses regarding the biochemical machinery underlying an observed phenotype. This utility of mgPipe has been demonstrated in various studies of the role of the human microbiome in complex conditions such as preterm birth, inflammatory bowel disease, colorectal cancer, and Parkinson’s disease (Baldini et al., 2020; Heinken et al., 2019; Hertel et al., 2019, 2021; Kindschuh et al., 2023). However, and despite its wide use and utility, only a MATLAB implementation of mgPipe is currently available, limiting its accessibility for those who are not proficient in MATLAB or cannot afford its license. Here, we provide a reliable, tested, open-source, and efficient Python implementation of mgPipe.

## Implementation & Availability

pymgpipe is a Python implementation of mgPipe (Baldini et al., 2019). It utilizes COBRApy (Ebrahim et al., 2013) as its main constraint-based metabolic modeling interface, and optlang (Jensen et al., 2017) to formulate and modify the underlying mathematical optimization problem. pymgpipe merges individual GEMs into a single model following mgPipe’s biologically-informed metabolic assumptions, such as the use of preordained diets, compartmentalized structure, abundance-scaled constraints on microbial flux contributions (Heinken et al., 2013), and community biomass optimization objective (Baldini et al., 2019). After building community-level models, metabolic profiles are computed in the form of NMPCs, as discussed above (Baldini et al., 2019). As part of this step, pymgpipe uses the VFFVA C package for a fast and efficient FVA implementation (Guebila, 2020). pymgpipe is compatible with both the Gurobi (Gurobi Optimization, LLC, 2023) and ILOG Cplex (IBM, Inc., 2023) solvers, which are both commercially available and free for academic use.

pymgpipe models are backwards-compatible with the MATLAB mgPipe models to ensure cross-software compatibility. Additionally, pymgpipe offers multithreading capabilities for both model construction and simulation, making it scalable to studies with a large sample size. The pymgpipe python package, as well as all associated documentation, tests, and example workflows, can be found at https://github.com/korem-lab/pymgpipe.

## Comparison to mgPipe

**Figure 1:**
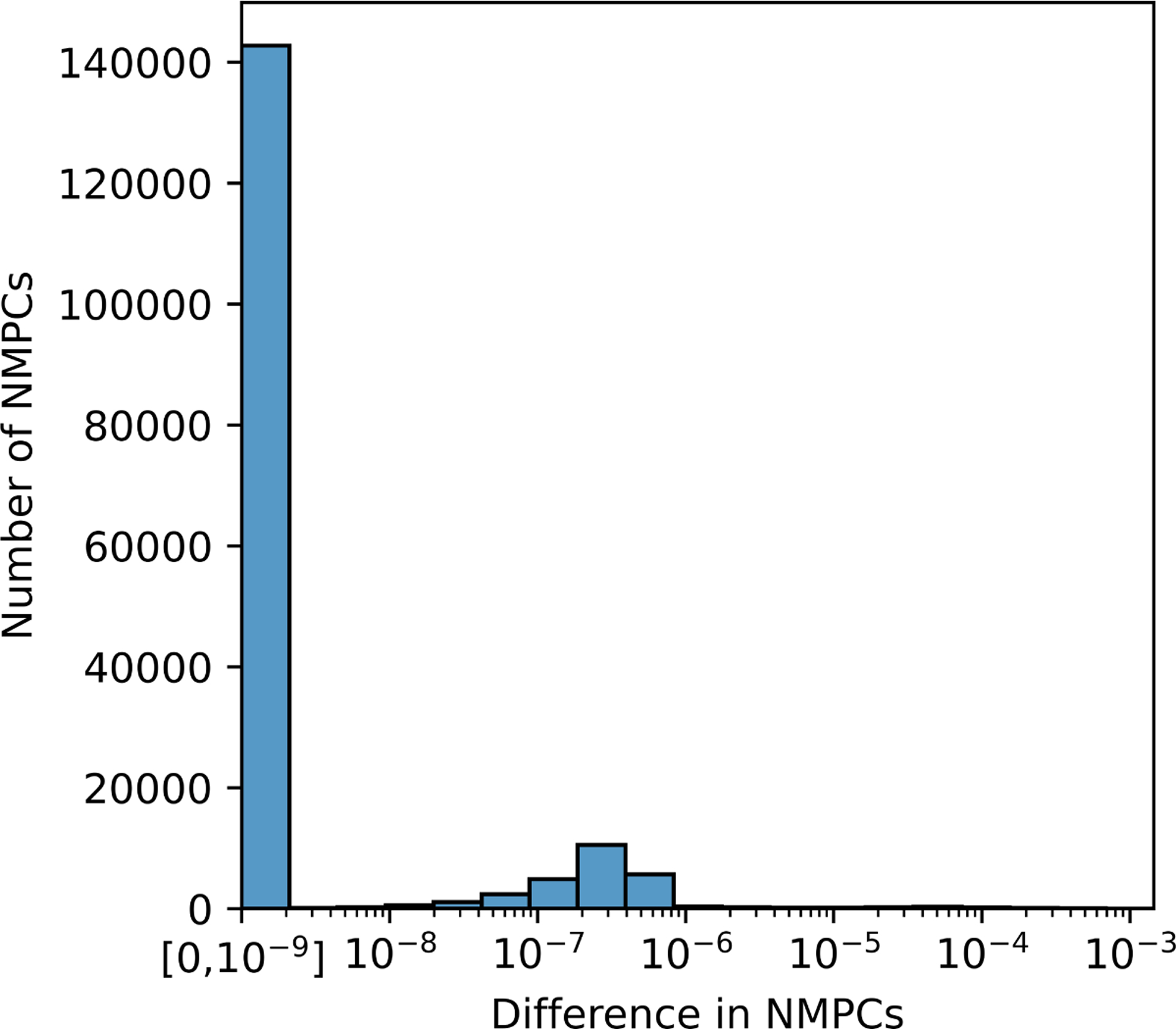
Histogram of magnitude of differences in NMPCs between mgPipe and pymgpipe.

To assess the accuracy of pymgpipe we compared its models and predictions with mgPipe, as implemented in the Microbiome Modeling Toolbox, Cobra Toolbox commit: 71c117305231f77a0292856e292b95ab32040711 (Baldini et al., 2019). We generated community-scale models for a vaginal microbiome dataset consisting of 232 samples, each composed of between 2 to 50 taxa (94 unique taxa), as previously described (Kindschuh et al., 2023). The models exhibited identical metabolic networks and structure between the two implementations (not shown). Additionally, metabolic profiles (NMPCs) output by pymgpipe exhibited only minor differences (mean±sd. 5.37e-7±1.23e-5; difference is below 1e-5 for 99.4% of all data points; Figure 1). These differences are negligible (within solver tolerance) and are most likely due to variations in FVA implementations (Guebila, 2020), solver versions, and tolerances. Overall, pymgpipe presents as an accurate Python implementation of the mgPipe pipeline.
